# Gasometric gradients between blood obtained from the pulmonary artery wedge and pulmonary artery positions in pulmonary arterial hypertension

**DOI:** 10.1186/s12931-018-0969-7

**Published:** 2019-01-08

**Authors:** Ghaleb Khirfan, Mostafa K. Ahmed, Michael D. Faulx, Wael Dakkak, Raed A. Dweik, Adriano R. Tonelli

**Affiliations:** 1 Department of Pulmonary, Allergy and Critical Care Medicine, Respiratory Institute, Cleveland Clinic, 9500 Euclid Avenue A-90, Cleveland, OH 44195 USA; 2 Department of Chest Diseases, Faculty of Medicine, Assiut University, Assiut, Egypt; 3 Department of Cardiovascular Medicine, Heart and Vascular Institute, Cleveland Clinic, Cleveland, OH USA; 4 Department of Internal Medicine, John H. Stroger Jr. Hospital of Cook County, Chicago, IL USA

**Keywords:** Blood gas analysis, Methemoglobin, Pulmonary hypertension

## Abstract

**Introduction:**

Little is known on the pulmonary gradients of oxyhemoglobin, carboxyhemoglobin and methemoglobin in pulmonary arterial hypertension (PAH). We sought to determine these gradients in group 1 PAH and assess their association with disease severity and survival.

**Methods:**

During right heart catheterization (RHC) we obtained blood from pulmonary artery (PA) and pulmonary artery wedge (PAW) positions and used co-oximetry to test their gasometric differences.

**Results:**

We included a total of 130 patients, 65 had group 1 PAH, 40 had pulmonary hypertension (PH) from groups 2–5 and 25 had no PH during RHC. In all groups, PAW blood had higher pH, carboxyhemoglobin and lactate as well as lower pCO_2_ than PA blood. In group 1 PAH (age 58 ± 15 years, 72% females), methemoglobin in the PAW was lower than in the PA blood (0.83% ± 0.43 vs 0.95% ± 0.50, *p* = 0.03) and was directly associated with the degree of change in pulmonary vascular resistance (*R* = 0.35, *p* = 0.02) during inhaled nitric oxide test. Oxyhemoglobin in PA (HR (95%CI): 0.90 (0.82–0.99), *p* = 0.04) and PAW (HR (95%CI): 0.91 (0.84–0.98), *p* = 0.003) blood was associated with adjusted survival in PAH.

**Conclusions:**

Marked differences were observed in the gasometric determinations between PAW and PA blood. The pulmonary gradient of methemoglobin was lower in PAH patients compared to controls and a higher PAW blood methemoglobin was associated with a more pronounced pulmonary vascular response to inhaled nitric oxide. Pulmonary artery and PAW oxyhemoglobin tracked with disease severity and survival in PAH.

## Introduction

Pulmonary arterial hypertension (PAH) is a condition characterized by progressive narrowing of the small pulmonary arteries that if left untreated leads to right heart failure and death [1]. This pre-capillary involvement results in a distinct hemodynamic profile characterized by a mean pulmonary artery pressure (mPAP) ≥ 25 mmHg, pulmonary artery wedge pressure (PAWP) ≤ 15 mmHg and pulmonary vascular resistance (PVR) > 3 Wood units [2]. During the last three decades, the understanding of the pathobiology of PAH has markedly improved; however, there remains a need to determine whether certain gasometric alterations play a role in the pathogenesis of PAH.

The lungs oxygenate the blood and remove the carbon dioxide (CO_2_) generated by metabolic processes. It remains unknown how the lungs of patients with PAH process carboxyhemoglobin (COHb) and methemoglobin (metHb); which are compounds potentially involved in the pathogenesis of the disease [3–6]. Endogenously, carbon monoxide (CO) is produced by the catabolism of heme by heme oxygenase [7]. Carbon monoxide is in part produced in the lungs [8, 9] where it may act as a pulmonary vasodilator [10]. Methemoglobin results from the oxidation of the hemoglobin iron to a ferric (Fe^+++^) state, a reaction that occurs when oxyhemoglobin reacts with nitric oxide (NO) [11]; therefore levels of metHb may track with levels of NO, a potent vasodilator implicated in the pathogenesis of PAH [12].

**Fig. 1:**
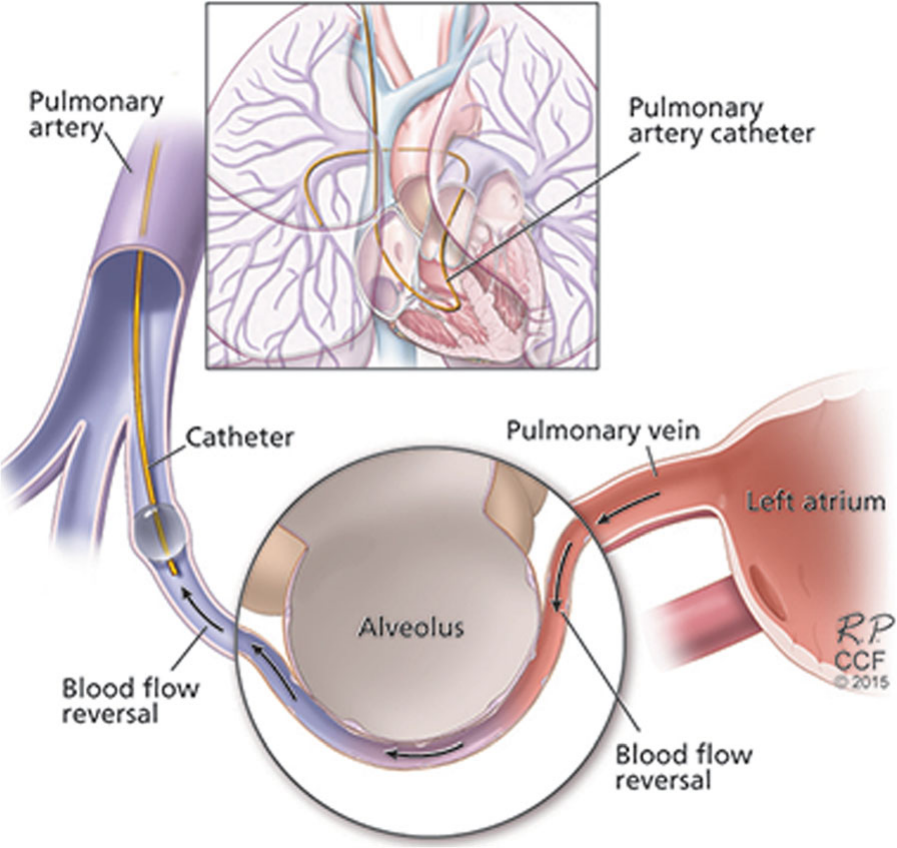
Illustration showing the origin of the pulmonary artery wedge blood during right heart catheterization

Transpulmonary gradients are traditionally measured between pulmonary artery (PA) (mixed venous) and systemic arterial blood (e.g. radial artery) [13–18]; however, arterial blood may be affected by right-to-left shunts from the Thebesian veins and potential metabolic processes between the left heart and the arterial sampling site. An approach to prevent these problems is to obtain blood from the pulmonary artery wedge (PAW) position (Fig. 1), indeed PAW blood has been studied in a variety of diseases [19–21]. Notably, PAW blood can be obtained at the time of right heart catheterization (RHC) without the need for an arterial puncture. In our practice we routinely obtain PA blood (mixed venous) for indirect/direct Fick cardiac output calculation and PAW blood to support an adequate PAW measurement [22]. Given that there is limited information on the pulmonary gradients of certain gases in PAH, we used co-oximetry to test the differences between blood obtained at the PA and PAW positions. We hypothesize that patients with PAH (group 1) have derangements in COHb and metHb as a result of defects in the NO pathway. We further hypothesize that COHb and metHb gradients may not only provide an insight on the metabolic processes that occur in the lungs of PAH patients but may be associated with disease severity, inhaled NO response and survival.

## Materials and methods

### Subjects and study design

This cross-sectional study was approved by the Cleveland Clinic institutional review board (study number: 06–245 and 14–1069). Written informed consent was waived given the retrospective nature of the study. We included consecutive patients who underwent RHC either to a) diagnose pulmonary hypertension (PH) or b) manage patients with prior diagnosis of PH. Right heart catheterizations were performed between December 2012 and April 2015.

We carefully assessed each patient to determine the etiology of PH based on the Fifth World Symposium classification [23]. Group 1 PH includes the idiopathic and heritable forms, PH associated with drugs, toxins, connective tissue and congenital heart diseases, portal hypertension, HIV infection and schistosomiasis. Group 2, 3, 4 and 5 includes PH due to left heart disease, lung disease/hypoxia, chronic thromboembolic disease and multifactorial mechanisms, respectively. For the purpose of this study, patients were divided into three groups: a) patients with PAH (PH group 1), 2) patients with PH groups 2–5, and 3) age- and gender-matched subjects without evidence of resting PH on RHC (mPAP < 25 mmHg). Patients without PH underwent RHC due to elevated right ventricular systolic pressure on echocardiography. Patients were excluded (*n* = 50) if the PAW blood could not be obtained during RHC or if it showed an SO_2_ < 90%, suggestive of an inadequate balloon wedging with leakage of deoxygenated blood from the proximal (before the balloon) to the distal (after the balloon) aspect of the PA catheter.

### Right heart catheterization

Subjects underwent RHC in the outpatient setting by a single operator (A.R.T.). All RHC were done under local anesthesia using 5 mL of 2% lidocaine. We continued or started oxygen (O_2_) supplementation in patients in whom the resting pulse oxygen saturation (SpO_2_) was < 90%. We maintained the same O_2_ flow during all the study measurements. In the supine position and with the transducer located in the mid-thoracic line (4th intercostal space), we measured the right atrial (RA) pressure, mean PAP and PAWP at end-expiration. We determined cardiac output (CO) by thermodilution. We calculated the cardiac index (CO / body surface area), the transpulmonary pressure gradient (TPG: mean PAP - PAWP), diastolic pulmonary gradient (DPG: diastolic PAP – PAWP) and PVR (TPG / CO). When appropriate, we performed a pulmonary vasodilator challenge using inhaled NO at 40 ppm for 5 min [24].

### Laboratory determinations

Pulmonary artery and PAW blood were obtained immediately after fluoroscopic site confirmation and obtaining an adequate waveform. The tip of the pulmonary artery catheter was located in West zone 3 [25]. Ten mL of blood were discarded from each site before a sample was collected in a blood gas syringe. Blood gas samples were immediately analyzed using the ABL 800 Flex analyzer (Radiometer, Copenhagen, Denmark) which uses co-oximetry and provide determinations of COHb, metHb, Hb and lactate.

### Other measurements

We collected data regarding demographics, use of PAH-specific medications and smoking status (current smoker was defined as a person who smoked tobacco in the last 30 days). We determined the severity of PH by using the New York Heart Association (NYHA) functional class, plasma N- terminal pro-B type natriuretic peptide (NT-proBNP), distance walked in the six-minute walk test (6MWD), RV size and function on echocardiography and hemodynamic determinations during RHC. Right ventricular function was determined both subjectively by visual inspection and objectively by the tricuspid annular plane systolic excursion (TAPSE) [26]. We also recorded the diffusion lung capacity for carbon monoxide corrected for Hb (DLCOc) [27].

### Statistical analysis

Continuous data are presented as mean ± standard deviation (SD) or median (interquartile range (IQR)) as appropriate. Categorical data are summarized as discrete values and percentages (n (%)). Continuous and categorical variables were compared across the groups using analysis of variance (ANOVA) and Chi-square, respectively. Paired data were contrasted with paired t test or Friedman test as appropriate. Associations were tested using the Pearson correlation test. Survival analysis was performed with Cox proportional hazards model adjusted by age, gender and other pre-specified variables. The starting point for the survival analyses was the date of the gasometric determinations. Patients were censored at the time of lung transplantation and followed until death or end of the study in January 2018. Cox proportional hazards model results are expressed as hazard ratios (HR) with the corresponding 95% confidence intervals (CI). All *p* values are two-tailed and a value of < 0.05 was considered significant. The statistical analyses were performed using the statistical package IBM SPSS, version 20 (IBM; Armonk, New York) and MedCalc, version 14.12.0 (Ostend, Belgium).

## Results

### Baseline characteristics

We included a total of 130 patients, of whom 65 had group 1 PAH, 40 had PH from groups 2–5 and 25 had no PH during RHC. Of the patients with PAH, 38 (58%) had idiopathic or heritable PAH, 17 (26%) had PAH associated with connective tissue diseases, 5 (8%) had porto-pulmonary hypertension and 5 (8%) had PAH due to other etiologies. Patients with non-group 1 PH belonged to PH groups 2 (*n* = 20, 50%), 3 (*n* = 10, 25%), 4 (*n* = 5, 12.5%) and 5 (n = 5, 12.5%). All patients without PH (*n* = 25, 19%) had an elevated RVSP (≥ 40 mmHg) and associated diseases such as scleroderma, cirrhosis, interstitial lung disease, obstructive sleep apnea or suggestion of left ventricular diastolic dysfunction by echocardiogram.

Of the patients with PAH, 18 (28%), 15 (23%), 20 (31%) and 12 (19%) were on none, 1, 2, and 3 PAH-specific therapies, respectively. These PAH-specific therapies were phosphodiesterase-5 inhibitors (*n* = 39, 60%), endothelin receptor antagonists (*n* = 25, 39%), soluble guanylate cyclase stimulator (*n* = 1, 2%), and prostacyclin analogues (*n* = 26, 40%). Baseline characteristics of the three groups of patients are shown in Table 1.

**Table 1:** Baseline patient characteristics

Variable	Group 1 pulmonary arterial hypertension	Groups 2–5 pulmonary hypertension	Control group	P (ANOVA / Chi square)
n (%)	65 (50.0)	40 (30.8)	25 (19.2)	
Age (years)	58 ± 15	59 ± 20	54 ± 20	0.52
Female gender, n (%)	47 (72)	22 (55)	15 (60)	0.17
NYHA functional class, n (%)				
I	5 (8)	4 (10)	6 (24)	0.14
II	26 (40)	10 (25)	9 (36)	
III	31 (48)	22 (55)	10 (40)	
IV	3 (5)	4 (10)	0 (0)	
Supplemental O_2,_ n (%)	29 (45)	21 (53)	6 (24)	0.07
FiO_2_ in patients on supplemental O_2_ (%)	42 ± 22	35 ± 16	29 ± 3	0.21
NT-proBNP (pg/mL)	2084 ± 3247	2087 ± 2737	180 ± 153	0.06
6MWD (m)	310 ± 129	317 ± 106	343 ± 130	0.61
DLCOc (% predicted)	54 ± 15	51 ± 28	60 ± 26	0.43
Echocardiography				
TAPSE (cm)	1.90 ± 0.62	1.81 ± 0.62	2.68 ± 0.80	0.45
RVSP (mm Hg)	73 ± 26	63 ± 22	50 ± 18	0.001
RHC				
RA pressure (mmHg)	8 ± 6	9 ± 5	4 ± 3	0.001
mPAP (mmHg)	42 ± 14	37 ± 10	17 ± 5	< 0.001
PAWP (mmHg)	10 ± 5	15 ± 6	8 ± 3	< 0.001
TPG (mmHg)	32 ± 13	23 ± 10	9 ± 3	< 0.001
CI (L/min/m^2^)	3.0 ± 0.9	2.7 ± 0.8	3.2 ± 0.7	0.06
PVR (Wood units)	6.7 ± 4.2	5.0 ± 3.5	1.5 ± 0.8	< 0.001
Gasometric determinations (PA)				
pH	7.41 ± 0.03	7.39 ± 0.04	7.40 ± 0.03	0.06
pCO_2_ (mmHg)	41.8 ± 5.7	47.7 ± 8.5	44.6 ± 4.6	< 0.001
SO_2_ (%)	68.1 ± 7.6	66.6 ± 9.7	72.4 ± 5.9	0.02
COHb (%)	1.6 ± 1.1	1.6 ± 0.9	1.4 ± 1.0	0.68
metHb (%)	1.0 ± 0.5	0.9 ± 0.4	0.8 ± 0.4	0.22
Lactic acid (mmoL/L)	0.8 ± 0.4	1.1 ± 0.5	1.0 ± 0.4	0.008
Gasometric determinations (PAW)				
pH	7.54 ± 0.08	7.49 ± 0.10	7.49 ± 0.08	0.02
pCO_2_ (mmHg)	27.1 ± 8.2	34.0 ± 10.6	32.3 ± 7.4	0.001
SO_2_ (%)	93.6 ± 5.4	92.0 ± 7.0	93.4 ± 3.6	0.38
COHb (%)	1.8 ± 1.2	1.7 ± 1.1	1.6 ± 1.0	0.88
metHb (%)	0.8 ± 0.4	0.8 ± 0.4	0.9 ± 0.4	0.84
Lactic acid (mmoL/L)	0.9 ± 0.4	1.2 ± 0.6	1.1 ± 0.5	0.04

*Definition of Abbreviations*: *COHb*: carboxyhemoglobin, *CI*: cardiac index, *DLCOc*: diffusion lung capacity for carbon monoxide corrected for hemoglobin, *FiO*_*2*_: fraction of inspired oxygen, metHb: methemoglobin, *mPAP*: mean pulmonary artery pressure, *NT-proBNP*: N-terminal pro-B type natriuretic peptide, *NYHA*: New York Heart Association, *PA*: pulmonary artery, *PAW*: pulmonary artery wedge, *PAWP*: pulmonary artery wedge pressure, *pCO2*: partial pressure of carbon dioxide, *PVR*: pulmonary vascular resistance, *RA*: right atrial, *RHC*: right heart catheterization, *RVSP*: right ventricular systolic pressure, SO_2_:oxygen saturation, TAPSE: tricuspid annular plane systolic excursion, *TPG*: transpulmonary pressure gradient, 6MWD: distance walked in six-minute walk test

Data are expressed as mean ± SD or n (%)

### Comparison of PAW and PA blood in patients with group 1 PAH

We observed significant differences between the PAW and PA blood in PAH patients. PAW blood had higher pH, COHb and lactate as well as lower pCO_2_, bicarbonate and metHb when compared to the PA blood (Table 2).

**Table 2:** Comparison of pulmonary artery wedge and mixed venous blood in pulmonary arterial hypertension patients (*n* = 63)

Variable	Pulmonary artery wedge blood	Mixed venous blood	Mean difference	95% CI of the difference	P (paired T test)
pH	7.53 ± 0.08	7.40 ± 0.03	0.13	0.11 to 0.15	<0.001
pCO_2_ (mmHg)	27.1 ± 8.2	41.9 ± 5.7	− 14.80	−16.42 to − 13.10	<0.001
pO_2_ (mmHg)	104.4 ± 53.5	39.6 ± 4.7	64.85	51.40 to 78.30	<0.001
pO_2_^a^ (mmHg)	82.5 ± 24.9	40.1 ± 4.8	42.48	38.01 to 50.96	<0.001
HCO_3_- (mmoL/L)	22.3 ± 4.0	25.7 ± 3.3	−3.39	− 3.83 to − 2.96	<0.001
O_2_Hb (%)	93.6 ± 5.4	68.3 ± 7.3	25.28	22.95 to 27.61	<0.001
O_2_Hb ^a^ (%)	92.7 ± 6.5	70.5 ± 6.3	22.20	19.11 to 25.28	<0.001
COHb (%)	1.76 ± 1.18	1.59 ± 1.1	0.16	0.06 to 0.26	0.002
COHb ^b^ (%)	1.52 ± 0.79	1.33 ± 0.61	0.16	0.07 to 0.24	<0.001
metHb (%)	0.83 ± 0.43	0.95 ± 0.50	− 0.12	− 0.23 to − 0.01	0.03
Hb (g/dL)	12.7 ± 2.4	12.8 ± 2.3	− 0.11	− 0.29 to + 0.07	0.24
Lactate (mmoL/L)	0.94 ± 0.35	0.82 ± 0.36	0.12	0.08 to 0.15	<0.001

*Definition of Abbreviations*: *CI*: confidence interval, *COHb*: carboxyhemoglobin, *Hb*: hemoglobin, *HCO*_*3*_-: bicarbonate, metHb: methemoglobin, *O*_*2*_*Hb*: oxyhemoglobin, *pCO*_*2*_: partial pressure of carbon dioxide, *pO*_*2*_: partial pressure of oxygen

Data are expressed as mean ± SD

^a^Patients not on Oxygen supplementation (*n* = 36)

^b^nonsmokers patients only (*n* = 60)

### Comparison of PAW and PA blood gradients among study groups

A comparison among the three study groups (group 1 PAH, non-group 1 PH and no PH) showed that the pH increase in the PAW compared to the PA blood was more pronounced in group 1 PAH patients (Table 3). We also noted that the metHb was lower in the PAW relative to PA blood in patients with PH (group 1 PAH and non-group 1 PH) compared to individuals without PH (Table 3). In fact, in individuals without PH, metHb was higher in the PAW than in the PA blood (mean (95% CI) difference: 0.12 (+ 0.01, + 0.23), *p* = 0.04).

**Table 3:** Differences between pulmonary artery wedge and mixed venous blood in the three groups of patients

Variable	Group 1 pulmonary arterial hypertension	Groups 2–5 pulmonary hypertension	Control group	P (ANOVA / Chi square)
n (%)	65 (50.0)	40 (30.8)	25 (19.2)	
pH	0.13 ± 0.07	0.10 ± 0.09	0.09 ± 0.06	0.05
pCO_2_ (mmHg)	− 14.7 ± 6.6	−14.2 ± 8.0	−12 ± 6.0	0.34
pO_2_ (mmHg)	64.9 ± 53.4	58.0 ± 47.4	42.7 ± 27.0	0.14
pO_2_^a^ (mmHg)	42.5 ± 24.7	36.4 ± 21.3	40.9 ± 26.5	0.70
HCO_3_- (mmoL/L)	− 3.4 ± 1.7	−3.0 ± 1.4	− 2.9 ± 1.3	0.33
O_2_Hb (%)	25.3 ± 9.3	23.7 ± 9.6	21.0 ± 6.3	0.12
O_2_Hb ^a^ (%)	22.2 ± 9.0	25.1 ± 6.7	21.9 ± 6.1	0.39
COHb (%)	0.16 ± 0.40	0.09 ± 0.41	0.21 ± 0.53	0.55
COHb ^b^ (%)	0.17 ± 0.41	0.09 ± 0.42	0.22 ± 0.56	0.55
metHb (%)	−0.12 ± 0.42	−0.11 ± 0.43	0.12 ± 0.27	0.04
Hb (g/dL)	− 0.11 ± 0.74	−0.19 ± 0.50	0.10 ± 0.61	0.20
Lactate (mmoL/L)	0.12 ± 0.13	0.10 ± 0.14	0.06 ± 0.08	0.12

*Definition of Abbreviations*: *CI*: confidence interval, *COHb*: carboxyhemoglobin, *Hb*: hemoglobin, *HCO*_*3*_-: bicarbonate, *metHb*: methemoglobin, *O*_*2*_*Hb*: oxyhemoglobin, *pCO*_*2*_: partial pressure of carbon dioxide, *pO*_*2*_: partial pressure of oxygen

Data are expressed as mean ± SD

^a^Patients not on Oxygen supplementation

^b^nonsmokers patients only

### Association between blood gases, gradients and disease severity in group 1 PAH patients

The gradient of O_2_Hb between PAW and PA was directly associated with RA pressure (R: 0.34, *p* = 0.006), PVR (R: 0.35, *p* = 0.006) and NT-proBNP (R: 0.36, *p* = 0.03) and inversely associated with CI (R: − 0.43, *p* < 0.001) and TAPSE (R: -0.32, *p* = 0.013). Other gasometric gradients were not significantly associated with markers of disease severity.

### Response to inhaled NO challenge and gasometric determinations in group 1 PAH patients

In group 1 PAH patients: the PVR decreased a mean of 16.3% ± 16.9% (*n* = 45). The percentage drop in PVR was inversely associated with Hb (*R* = − 0.34, *p* = 0.02) and directly associated with metHb (*R* = 0.35, *p* = 0.02) and O_2_Hb (*R* = 0.321, *p* = 0.03) in the PAW blood.

### Relationship between blood determinations, gradients and survival in PH patients

The median (IQR) follow-up for patients with type 1 PAH was 41 (22–51) months, with a 2-year survival of 78%. When adjusted for age and gender, variables associated with mortality in group 1 PAH included: PA O_2_Hb (HR (95%CI): 0.87 (0.81–0.94), *p* = 0.001) and PAW O_2_Hb (HR (95%CI): 0.93 (0.87–0.98), *p* = 0.01). Pulmonary artery O_2_Hb was associated with mortality even when adjusted by supplemental oxygen therapy (HR (95%CI): 0.89 (0.82–0.96), *p* = 0.003) and adding PVR, 6MWD and number of PAH-specific therapies (HR (95%CI):0.90 (0.82–0.99), *p* = 0.04). Pulmonary artery wedge O_2_Hb continued to be a significant predictor when adjusted by supplemental oxygen therapy (HR (95%CI): 0.89 (0.83–0.96), *p* = 0.001), PA O_2_Hb (HR (95%CI): 0.88 (0.82–0.95), *p* = 0.001), and adding PVR, 6MWD and number of PAH-specific therapies (HR (95%CI):0.91 (0.84–0.98), *p* = 0.003). Methemoglobin or COHb levels measured in the PA or PAW, or their pulmonary gradients, did not predict survival.

## Discussion

In the present study we reported results on gasometric gradients between PAW and PA blood and compared findings in patients with group 1 PAH, PH groups 2–5 and age- and gender-matched disease controls. In all groups, we noted that the PAW blood showed higher pH, COHb and lactate compared to the PA blood. Interestingly, the level of metHb was lower in PAW blood than in PA blood in patients with PH, in contrast to higher levels in the PAW blood in patients without PH. In patients with group 1 PAH, we noted that a higher PAW metHb was associated with a more pronounced PVR drop during inhaled nitric oxide challenge. In patients with group 1 PAH, a lower PA and PAW O_2_Hb levels were independently associated with worse survival.

As shown by others [19, 20], the pH was higher in the PAW blood compared to the PA blood, in association with a lower pCO_2_. This pH gradient was more pronounced in patients with PAH than the other 2 groups of patients. Patients with PAH tend to have lower arterial pCO_2_ [28, 29] given an increase in the respiratory drive, an effect that becomes more evident in the PAW blood. Interestingly, the levels of lactate were higher in the PAW than PA blood suggesting lung production, as seen in other diseases such as acute respiratory distress syndrome [14, 30, 31]. We speculate that the differences in PAW blood are due to a) double lung passage of blood, initially from the PA through the alveolar capillaries and pulmonary veins to the left atrium, and then backwards from the left atrium through the pulmonary veins and alveolar capillaries to the PA catheter, leading to longer exposure to alveolar gas and intensification of the lung processes (Fig. 1) and/or b) prolonged exposure of the PAW blood to alveolar gases, given the temporal immobility of blood in a functional portion of the lung.

Nitric oxide is a potent vasodilator [32] that plays an important role in the pathogenesis of PH [12]. The reaction with O_2_Hb is one of the catabolic pathways for NO, a process that generates nitrate and metHb [33]. Naples et al. showed that venous metHb correlated with the levels of NO in the plasma of asthmatic patients [34]. In our study, we showed that blood metHb levels are lower in the PAW compared to PA position in PH patients; in contrast, we found higher levels of metHb in PAW position in patients without PH. These findings insinuate a reduced production or an enhanced metabolism of metHb in the lung of PH patients. Interestingly, in PAH patients, a higher metHb in PAW blood was associated with a more pronounced decline in PVR during inhaled NO challenge, suggesting a greater diffusion of NO, a healthier pulmonary vasculature and/or an earlier stage of the disease in these patients. Further investigations may determine whether the level of metHb in the PAW position is associated with the response to PH-medications that target the nitric oxide pathway.

Carboxyhemoglobin is produced endogenously during the oxidation of heme compounds by heme oxygenase [35]. The carbon monoxide released from this catabolic reaction has vasodilatory [36], anti-inflammatory [37] and anti-proliferative properties [4]. Hypoxia transiently increases expression of heme oxygenase [38, 39]. The increased expression of heme oxygenase prevented the development of PH induced by hypoxia [38, 39] a mechanism that may involve the antiproliferative action of carbon monoxide [39]. In the present study, we found that COHb is higher in the PAW compared to PA blood in all groups of patients, suggesting pulmonary production and systemic metabolism of carbon monoxide. A finding consistent with prior studies which showed positive arteriovenous COHb difference, with higher COHb in arterial compared to venous blood [8, 34, 40].

Analysis of survival in group 1 PAH patients showed that PA and PAW O_2_Hb adjusted by age, gender, use of supplemental oxygen, PVR and 6MWD are independent predictors of long-term survival. The association between PA O_2_Hb and survival was expected given previous research [41, 42]. The association between PAW blood O_2_Hb and survival is novel and consistent with our prior study showing that hypoxemia (determined by pulse oximetry) in patients with idiopathic and heritable PAH is associated with worse survival [43].

Our study has limitations: a) retrospective analysis, b) even though the gasometric differences of metHb are small and might not be clinically significant, they provide an insight on the complex physiological processes involving nitric oxide and c) although patients had a detailed smoking history, surreptitious smoking or other environmental exposure that increased carbon monoxide could not be ruled out. Smoking may affect the pulmonary endothelium, leading to pulmonary vascular remodeling and PH, even in the absence of hypoxemia and destructive emphysematous lung disease [44–48]. Despite these limitations, our study is the first to assess the gasometric gradients between PAW and PA blood in patients with PAH.

## Conclusions

Pulmonary artery wedge blood samples had higher pH and lower pCO_2_ than PA, suggesting that PAW blood was exposed to alveolar gas for a longer period of time. Methemoglobin levels were lower in the PAW than PA blood in patients with PAH and a higher PAW metHb was associated with a more pronounced pulmonary vascular response to inhaled NO. Pulmonary artery and PAW O_2_Hb tracked with disease severity and survival in PAH patients.
